# Thrombolytic Agents: Nanocarriers in Targeted Release

**DOI:** 10.3390/molecules26226776

**Published:** 2021-11-10

**Authors:** Minghua Shen, Yujiao Wang, Fan Hu, Linwen Lv, Kui Chen, Gengmei Xing

**Affiliations:** 1 Department of Biochemistry and Molecular Biology, Yanbian University Medical College, Yanji 133002, China; sdjjch@ybu.edu.cn; 2 CAS Key Laboratory for Biomedical Effects of Nanomaterial & Nanosafety, Institute of High Energy Physics, Chinese Academy of Sciences, Beijing 100049, China; wangyujiao@ihep.ac.cn (Y.W.); hufan@ihep.ac.cn (F.H.); lvlinwen@ihep.ac.cn (L.L.); 3 Key Laboratory for Molecular Enzymology and Engineering of the Ministry of Education, School of Life Sciences, Jilin University, Changchun 130012, China

**Keywords:** nano-drug delivery system, biological nano-drug delivery system, physical responsive nano-drug delivery system

## Abstract

A thrombus, known as a blood clot, may form within the vascular system of the body and impede blood flow. Thrombosis is the most common underlying pathology of cardiovascular diseases, contributing to high morbidity and mortality. However, the main thrombolytic drugs (urokinase, streptokinase, etc.) have shortcomings, including a short half-life, serious side effects and a lack of targeting, that limit their clinical application. The use of nano-drug delivery systems is expected to address these problems and a variety of approaches, including biological and physical responsive systems, have been explored. In this report, recent advances in the development of targeted nano-drug delivery systems are thoroughly reviewed.

## 1. Introduction

Blood plays indispensable roles that include the transportation of oxygen and nutrients, removal of waste products, regulation of body temperature and defense against infection. Under normal conditions, there is a balance between the blood coagulation and fibrinolytic systems that ensures blood fluidity and the potential for clotting. Disturbance of this equilibrium leads to blood coagulation, or agglutination of blood components, to form solid matter in the heart and blood vessels of mammals. This process is called thrombosis and the solid matter is known as a thrombus [1,2]. Thrombosis can occur in arterial or venous systems and has a significant impact on human health. There are three principal factors, Virchow’s triad, that cause venous thrombosis: venous stasis, vascular endothelial injury and hypercoagulability of blood [3]. In contrast, arterial thrombosis is highly dependent on the state of the vessel wall, platelets and factors related to blood flow. Consequently, thrombolytic agents used to treat arterial thrombosis are antiplatelet drugs, while drugs targeting clotting factors in the coagulation cascade are used to treat venous thromboembolism.

There are currently many types of thrombolytic drug used in the clinic. First-generation thrombolytic drugs, urokinase (UK) and streptokinase (SK), have short half-lives and are not targeted to fibrin. Second-generation (tPA) and third-generation agents (TNK-tPA and reteplase) exhibit an affinity for fibrin [4]. However, these drugs have disadvantages, such as low bioavailability, off-target side effects, limited penetration of thrombi and an increased risk of uncontrolled bleeding [5]. Systemic delivery and nonspecific activation of thrombolytic agents increase the likelihood of hemorrhage and limit the use of certain drugs. Furthermore, protein-based thrombolytic drugs are rapidly inactivated when injected into the bloodstream, resulting in the need for large doses [6]. The development of nano-technology has provided various types of nanoparticles to overcome the limitations of thrombolytic therapy. Targeted drug delivery to the clotting site is an attractive approach because it reduces side effects, increases drug stability and prolongs the half-life.

Recently, a variety of carriers have been considered for the controlled release of thrombolytic drugs, including mesoporous silica, liposomes, and magnetic nanoparticles and dendrimers. Many studies have demonstrated that nanocarriers can increase targeting, biological safety and the circulatory half-life, enabling a reduction of dosage and resulting in fewer side effects. For instance, nanoparticles can prevent direct contact between drug and blood to protect against degradation and maximize the therapeutic effect.

Importantly, one advantage of nanoparticle drug delivery is to enhance thrombolytic therapy by improving targeted release at the site of coagulation. Different modifications result in increased thrombolytic effects of nanoparticles due to various factors. Here, we thoroughly reviewed recent advances in the development of nanocarriers for targeted delivery.

## 2. Mechanisms of Thrombus Formation

### 2.1. Venous Thrombi

Under normal physiological conditions, cardiovascular endothelial cells have an anticoagulant effect [7]. In contrast, when vascular endothelial cells are damaged, their anticoagulant properties are disrupted. In general, damage to endothelial cells is the major reason for thrombosis formation. In compromised endothelial cells, collagen beneath the vascular endothelium is exposed, which activates platelets and enables the release of coagulation factor XII, promoting endogenous coagulation. Activation of platelets is pivotal in the process of thrombosis [8]. First, platelets adhere to the exposed collagen surface after injury of the intima. In response to collagen, the platelets are then switched from the resting state to the activated state. Upon activation, the shape of platelets changes from typically discoid to spherical with long projections that can extend microns from the cell body. The glycoproteins on the surface of the platelets are also altered. Expression of integrins α_5_β_1_ and α_6_β_1_ by platelets enables direct adherence to fibronectin and laminin in the extracellular matrix. Platelet endothelial cell adhesion molecule-1 (PECAM-1) mediates platelet adhesion to endothelial cells, some leukocytes and heparin [9]. P-selectin, a standard marker of platelet activation, plays a role in anchoring leukocytes (expressing L-selectin) with endothelial cells (expressing E-selectin and P-selectin glycoprotein ligand-1) [10]. Alongside the initiation of endogenous and exogenous coagulation pathways, thrombin converts fibrinogen to fibrin. Fibrin binds to fibronectin in the intimal matrix, so platelets and a few white and red blood cells become firmly fixed to the intima of damaged vessels.

The conditions for venous thrombosis are summed up in Virchow’s triangle theory: cardiovascular endothelial cell injury, changes in blood flow and hypercoagulability of blood [3]. In addition, white blood cells contribute to the formation of arteriovenous thrombosis. For example, neutrophils release DNA fibers within the cell in an inflammatory environment, thus stimulating thrombosis [11].

#### 2.1.1. Injury of Cardiovascular Endothelial Cells

Cardiovascular endothelial cells have anticoagulant and procoagulant properties. Under normal physiological conditions, the main effect is anticoagulation. Intact endothelial cells separate coagulation-promoting platelets, clotting factors and the subcutaneous extracellular matrix. At the same time, prostacyclin and nitric oxide can be synthesized to inhibit platelet adhesion [12,13], and ADP enzyme can be secreted to convert ADP into adenine nucleotide to resist platelet adhesion [14]. The endothelial cell surface can also synthesize or express anticoagulant proteins and molecules, such as thrombin regulatory proteins and membrane-associated heparin-like molecules. The former can bind with thrombin in blood to activate protein C [15], which synergizes with protein S to inactivate factors V and VIII. Membrane-associated heparin-like molecules can bind to antithrombin III, inactivating clotting factors such as thrombin, factor X and factor IX. Endothelial cells also tend to consolidate the synthesis of tissue plasminogen activator, which is able to clear deposits of fibrin on the endothelial cell surface and maintain blood flow. When endothelial cells are damaged, the exposed subendothelial collagen and tissue factor released by endothelial cells will initiate intrinsic coagulation and extrinsic coagulation [16]. Meanwhile, von Willebrand factor will be released to assist platelet and collagen adhesion. In addition, endothelial cells can release inhibitors of plasminogen activator which restrain fibrinolysis [17].

#### 2.1.2. Changes in Blood Flow

Slowing of blood flow or vortex formation can promote thrombosis [18]. In normal blood flow, red blood cells and white blood cells are in the center of the blood flow, and platelets are in the outermost layer of plasma. The flow separates components of blood from the vessel wall. The main role of platelets is to contact and activate the inner membrane. When blood flow slows or whirlpools occur, platelets can enter the boundary layer flow, which increases the likelihood of contact and adherence to the inner membrane.

#### 2.1.3. Hypercoagulability of Blood

Increased numbers of platelets and coagulation factors in blood, or reduced activity of the fibrinolytic system, result in a high coagulation state [19].

### 2.2. Arterial Thrombi

The pathophysiological conditions of arterial thrombosis differ from those of venous thrombosis. Arterial thrombosis and atherosclerosis are closely related [20]. When unstable atherosclerotic plaques rupture or erode, matrix proteins, such as von Willebrand factor (vWF), fibrous collagen, fibronectin and laminin, are exposed to the blood. These proteins bind to platelets via special surface receptors [21,22]. Collagen binds to GPVI and integrin α_2_β_1_, laminin binds to integrin α_6_β_1_, fibronectin binds to integrin α_5_β_1_ [23], and vWF binds to GPIb-V-IX on the surface of platelets. Neither coagulation cascades nor Virchow’s triad mediate high shear rate thrombosis. At low shear rates (e.g., 50/s), clotting dominates thrombus formation, leading to red erythroid-rich thrombi. When the shear rate is above 5000/s, platelet accumulation dominates, resulting in white arterial clots. It has been shown that white clots form at higher fluid shear rates, while red clots form at very low shear rates [24]. Arterial thrombosis typically develops in an environment characterized by much higher shear stress and blood flow than in the venous system (i.e., 3.0–26 vs. 1.2–4.8 mL/min) [25].

## 3. Types of Thrombolytic Drugs

So far, three generations of thrombolytic drug have been developed for clinical use. The first-generation thrombolytic drugs were urokinase and streptokinase [26]. Streptokinase is a 47 kDa protein produced by the beta-hemolytic Streptococci species [27]. Its mechanism of action is via formation of a 1:1 complex with plasminogen, which induces a conformational change that exposes the active fibrinolytic site [28]. The enhanced fibrinolytic activity promotes dissolution of fibrin from thrombi. Because streptokinase is an exogenous protein, it stimulates antibody production in humans.

Urokinase is an endogenous trypsin-like hydrolase produced by human renal parenchymal cells that converts plasminogen into active plasmin [29]. Plasmin is a fibrinolytic enzyme that can directly degrade fibrin in a thrombus. Urokinase is rapidly degraded and cleared by the liver, with an average plasma half-life of 15 min. Because of its short half-life, re-embolization may occur within 15–30 min of stopping administration. Consequently, urokinase thrombolytic therapy is generally combined with heparin anticoagulation. Neither of the two first-generation thrombolytic drugs are targeted, either to fibrin or to the site of the thrombus.

Tissue plasminogen activator (tPA) is a second-generation thrombolytic therapy. Alteplase (rt-PA) is a recombinant form of the naturally occurring tPA [30]. In the presence of fibrin, tPA lyses the arginine-valine bond of plasminogen to form the serine protease, plasmin. In the absence of fibrin, however, this transformation is inhibited. Alteplase is widely used to control thrombosis, especially within 3 h of acute ischemic stroke, for which it is considered to be the most effective agent. Second-generation thrombolytic drugs have fibrin-targeting properties, but a mild or moderate reduction of circulating fibrinogen and plasminogen has been observed in clinical trials, together with some side effects [31].

The third-generation thrombolytic drugs, including TNK-tPA and reteplase, are the result of modifications to the structure of tissue plasminogen activator. TNK-tPA is obtained by replacements of threonine 103 of tPA with aspartic acid, aspartic acid 117 in the Kringle 1 domain with glutamine, and amino acids 296–299 with four alanine residues [32]. These modifications endow TNK-tPA with a longer blood half-life and higher fibrin binding specificity. Reteplase, with a molecular weight of 39 kDa, is a mutant of tissue plasminogen activator, lacking part of the amino acid sequence [33]. It is obtained commercially by expression in Escherichia coli. Compared with tPA, reteplase has lower affinity for fibrin but a longer blood half-life.

On the basis of previous reviews, we summarize the limitations of current thrombolytic drugs in Table 1, such as short half-life and off-target effect.

## 4. Targeting by Nanocarriers

**Figure 1:**
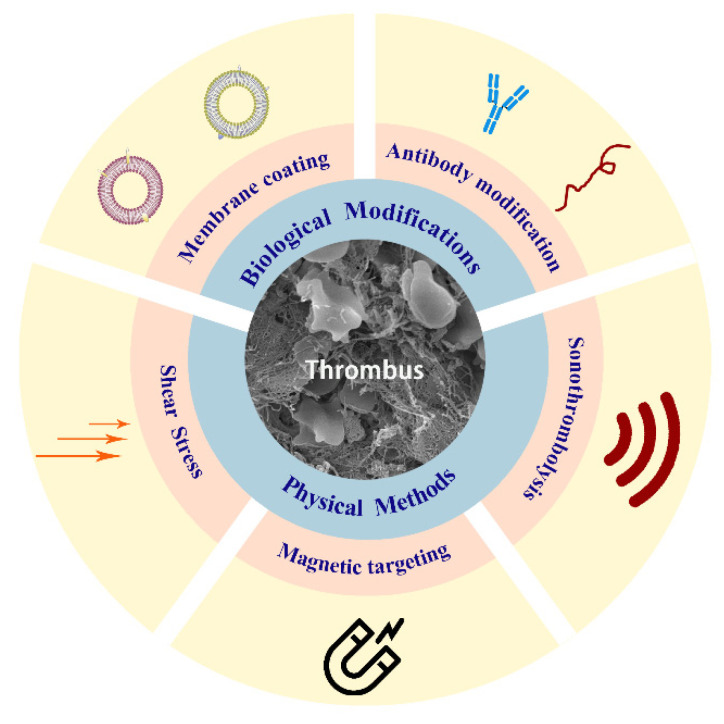
Schematic illustration of the main methods in modification of thrombolytic drug delivery nanocarriers.

A variety of vector targeting approaches have been proposed to control therapeutic drug release, including physical and biologically responsive targeting. Here, we summarize in Figure 1 the various approaches used for the delivery of thrombolytic drugs.

### 4.1. Physical Responsive Nano-Drug Delivery Systems

#### 4.1.1. Ultrasound-Mediated Drug-Loaded Thrombolysis System

Biomedical ultrasound (US) usually refers to sonic waves with frequencies higher than 20 kHz that can be absorbed and attenuated after being reflected or refracted by human tissues. The imaging acquisition system is responsible for recording the echoes and constructing ultrasonic images [38]. With the widespread application of ultrasound technology and the development of US contrast agents, ultrasound targeted microbubble destruction (UTMD) technology has made great progress for drug delivery and gene/anti-tumor/thrombolysis therapy via US contrast agents (US CAs). The US CAs are mainly used for contrast-enhanced (CE) US imaging, ultrasound cavitation, sonoporation and thermal ablation. 

**Figure 2:**
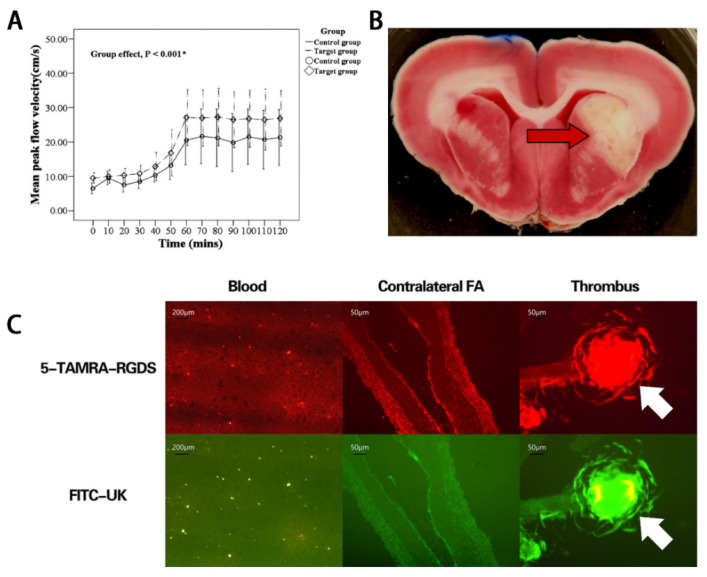
(**A**) Microbubbles combined with urokinase significantly improved blood flow (the urokinase plus microbubbles is the target group). (**B**) Detection of infarction area after middle cerebral artery occlusion. Axial section of rabbit brain stained with TTC and photographed for evaluation of infarction (arrow) size. Reproduced with permission [42]. Copyright 2018, Elsevier. (**C**) Fluorescence microscopy showed that the TAMRA- and FITC-conjugated microbubbles accumulated (yellow clumps) at the thrombus site. Reproduced with permission [43]. Copyright 2020, Nature publishing group.

Ultrasound can dissolve thrombi by mechanical means, such as acoustic cavitation, and accelerate vascular recanalization. Combined with microbubbles (MBs), ultrasonic cavitation and sonothrombolysis can be enhanced. Microbubbles are small, gas-filled microspheres, typically in the range of 1–8 µm diameter [39]. The ultrasound-mediated thrombolytic effect relies on the interaction between acoustic waves and microbubbles. The oscillating microbubbles can mechanically dissolve a thrombus [40]. In addition, a drug-loaded microbubble can be destroyed by ultrasound and then release drug specifically in the diseased region [41]. This can increase local drug concentrations and reduce toxic side effects compared with traditional drug treatment. Liu et al. designed a microbubble coated with urokinase to dissolve intracranial thrombi using low frequency ultrasound and consequently reduce the area of cerebral infarction (Figure 1B and Figure 2A) [42]. To improve the targeting of microbubbles, ligand modification, including avidin bridging, streptavidin and ligand, is one of the most common methods. In 2020, Guan et al. designed an ultrasonic MB carrying UK and an RGDS tetrapeptide (Arg-Gly-Asp-Ser) that could target thrombi and be activated by ultrasound. Their results demonstrated that US, targeted microbubbles, and urokinase synergized to achieve complete recanalization of the femoral artery in a rabbit model (Figure 2C) [43]. 

#### 4.1.2. Magnetic Targeted Nanoparticles

**Figure 3:**
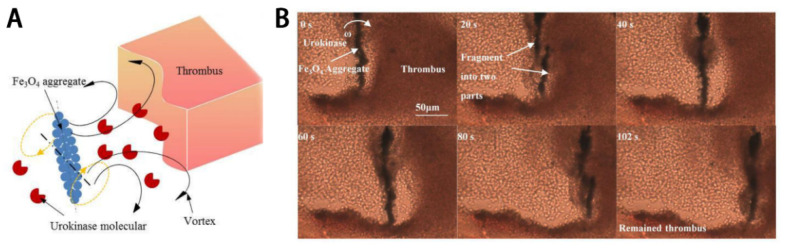
(**A**) The diffusion of urokinase is manipulated by the vortex induced by the rotation of rotating magnetic field (RMF)-guided aggregates. (**B**) Image sequences of thrombus removal by the RMF-guided Fe_3_O_4_ NPs. Reproduced with permission [47]. Copyright 2018, MDPI.

A thrombolytic drug delivery system based on magnetic particles can be targeted using an external magnetic field, allowing the particles to be directed down “blind alleys” in the vasculature, or even tangential to or against the prevailing flow, thereby improving drug delivery to the area of obstruction. Thrombolytic agents may be coated onto the surfaces of magnetic nanoparticles or encapsulated into nanoparticle polymeric shells, then transported to the target lesion under the control of an external magnetic field to release drug to dissolve the thrombus. Magnetic nanoparticles (MNPs) for thrombolytic therapy are prepared using FDA-approved materials with low toxicity to humans [44,45,46] and superparamagnetic iron oxide crystals, including magnetite (Fe_3_O_4_) or maghemite (γ-Fe_2_O_3_). Ming Chang manipulated surface-functionalized Fe_3_O_4_ nanoparticles coated with urokinase by means of magnetic fields for thrombolysis in a microfluidic channel. The average thrombolytic efficiency of magnetically-controlled urokinase-coated Fe_3_O_4_ nanoparticles increased by about 50% compared to free urokinase (Figure 3A,B) [47]. Similarly, silica-coated magnetic nanoparticles with a superparamagnetic iron oxide core (SiO_2_-MNP) were designed to improve clinical thrombolytic therapy. Compared with no magnetic targeting and free tPA, SiO_2_-MNP-tPA reduced blood clot lysis time by 34% and 40% in vitro [48].

When combined with magnetic materials, nanoparticles gathering around a thrombus can be observed using magnetic resonance imaging. In 2014, Zhou et al. constructed Fe_3_O_4_-based poly (lactic-co-glycolic acid) (PLGA) nanoparticles to detect and monitor early thrombosis. Coated with a film of CS (chitosan) or CS-cRGD, the nanoparticles (Fe_3_O_4_-PLGA-rtPA/CS-cRGD) had a significant effect on thrombolysis [49].

In a recent study, peptide/rtPA-conjugated PLGA magnetic nanoparticles (PPMNP-RTPA) were prepared by co-immobilization of rtPA and fibrin-avid peptide to PLGA magnetic nanoparticles. The dual-targeted PPMNP-RTPA reduced the time for dissolution of blood clots for reperfusion by 40% compared with free rtPA at the same dosage [50].

#### 4.1.3. Shear Stress Responsive Nanocarriers

Thrombotic vessels exhibit different physical characteristics compared with normal vessels. The shear stress of veins is usually 0.8–8 dyne/cm^2^ [51]. Fluid shear stress caused by highly constricted vessels can be locally increased by one to two orders of magnitude, from below ~70 dyne/cm^2^ in normal vessels to 1000 dyne/cm^2^ in highly constricted arteries [52,53,54,55]. A large number of reports have illustrated the ability of platelets to sense various mechanical forces and changes in mechanical force exposure [56]. The main factor in atherosclerotic plaque formation is local activation of circulating platelets by high shear stress, such that they rapidly adhere to these regions [57,58,59]. 

**Figure 4:**
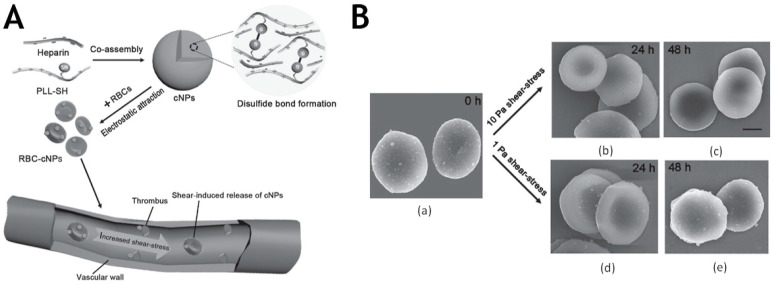
(**A**) Schematic illustration showing the preparation process of red blood cell adsorbed cNPs (RBC-cNPs) and the change of shear stress/rheology at the thrombus site can induce the release of cNPs. (**B**) SEM images of RBC-cNPs under 1 or 10 Pa shear-stress treatment for 24 and 48 h. (**a**) SEM image of RBC-adsorbed cNPs (RBC-cNPs) under static condition. SEM images of RBC-cNPs under 10 Pa shear-stress treatment for (**b**) 24 and (**c**) 48 h. SEM images of RBC-cNPs under 1 Pa shear-stress treatment for (**d**) 24 and (**e**) 48 h. Reproduced with permission [61]. Copyright 2016, Wiley.

Inspired by this natural physical mechanism of platelet targeting, the use of local high shear stress as a generic mechanism for targeting of clots, stenosis, or developmental abnormalities may become an effective treatment strategy. Netanel Korin and his colleagues designed micrometer-sized platelet bionic carrier (SA-NT) aggregates composed of small (180 ± 70 nm) nanoparticles [60]. It was demonstrated that SA-NTs were highly stable under normal blood flow conditions, but disintegrated to release drug at high shear stress sites. Chen et al. designed a co-assembly of heparin and polypeptide hybrid nanoparticles that attached to the surface of red blood cells. By exploiting the rapid change of shear stress at the site, hybrid nanoparticles were able to accurately release drug to treat the thrombus (Figure 4A,B) [61]. Margaret N. Holme synthesized a lenticular vesicle that formed a transient pore in constricted blood vessels with high shear stress and preferentially released drug [62]. The results suggested that carriers could be designed based on sharp rises of shear stress at the site of thrombosis to achieve precise release. 

### 4.2. Biologically Targeted Nano-Drug Delivery System

#### 4.2.1. Antibody or Peptide Modified Nanocarriers Based on Thrombus Pathophysiological Conditions 

Various nano-delivery carriers have emerged over recent decades, including highly successful liposomes. Liposomes have been studied for decades as drug nano-delivery carriers, and have advantages such as low cytotoxicity, good biocompatibility, simple preparation and easy surface modification [63,64]. The FDA has approved a number of liposome-based agents for use in antimicrobial and antitumor therapies, such as adriamycin and daunorubicin liposomes [65]. Liposomes also have great potential for thrombolytic therapy. Zhang et al. prepared liposomes modified with cyclic RGD peptide containing urokinase, which targeted the activation of platelets for the local release of urokinase, resulting in targeted thrombolysis, a prolonged half-life and a reduced bleeding risk [66]. Liposomes can also target P-selectin on the surface of activated platelets. Pawlowski et al. simultaneously conjugated the αIIbβ_3_-targeting polypeptide, GSSSGRGDSPA, and P-selectin polypeptide to the polyethylene glycol (PEG) termini of liposomes, achieving dual targeting of thrombosis activated platelets and improving the specificity and targeting efficiency [67]. Fucoidan, which has a nanomolar affinity for P-selectin expressed by activated platelets, was selected to target platelets and promote specific accumulation of loaded rtPA at the thrombus site [68]. Further examples of the different ligands used to decorate liposomes are summarized in Table 2.

#### 4.2.2. Cell Membrane-Coated Bionic Nanocarriers

**Figure 5:**
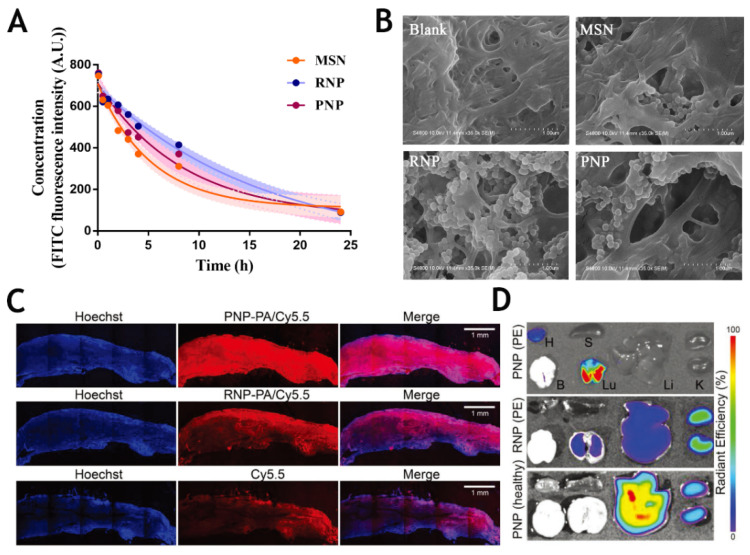
(**A**) The blood circulation time of the three nanoparticles (MSN/PNP/RNP) proved that the coating of erythrocyte membrane and platelet membrane can prolong the blood circulation time of nanoparticles to varying degrees. (**B**) RBC membrane (RNP) and platelet membrane (PNP) coated nanoparticles have different degrees of affinity for fibrin. Reprinted with permission from [75]. Copyright (2020) American Chemical Society. (**C**) Confocal images of patient thrombi. Nuclei were stained with Hoechst 33342 (blue), PNP-PA and RNP-PA were labeled with Cy5.5 (red). (**D**) Cy5.5-labeled nanoparticle (RNP/PNP) was significantly enriched in the lung of the PE model. H, heart; B, brain; S, spleen; Lu, lung; Li, liver; K, kidney. Reproduced with permission [76]. Copyright 2020, Wiley.

The traditional stealth functionalization strategy is to modify nanoparticles with polyethylene glycol (PEG) to extend their half-life, but it has been shown that PEGylation may lead to an anti-PEG immunological response. Alternative strategies, including synthetic polymers and biopolymers, have been developed, but these methods have resulted in only a limited improvement of histocompatibility. Apart from the traditional chemical decoration of nanoparticles, diverse nanoparticle coatings have been proposed. Cell membrane-coated nanoparticles have received increasing attention in distinct research areas. Cloaking nanoparticles with a natural membrane can prolong systemic circulation and enable cell-specific targeting [73]. Using a novel top-down strategy, natural cell membranes can be completely translocated to nanoparticles conferring the advantages of both synthetic and natural materials [74]. As a novel nanoparticle modification strategy, it has also been applied to thrombolytic therapy in recent years. Chen and colleagues demonstrated that erythrocyte membrane-coated mesoporous silica nanoparticles effectively prolonged the blood circulation time and exhibited superior targeting of fibrin (Figure 5A,B) [75]. Xu et al. designed a platelet bionic nanoparticle conjugated with rtPA using platelet membrane to improve biodistribution and achieve a clot-targeting thrombolytic therapy (Figure 5C,D) [76]. Moreover, it has been reported that cloaked platelet membrane PNPs loaded with lumbrokinase accumulated at sites containing fibrin, platelets and activated endothelial cells. The enrichment provided by nano-delivery systems has resulted in improved thrombolytic effects and a reduced risk of hemorrhage. Multiple key receptors involved in thrombosis (including integrin CD62p, GPVI and GPIbα) [77] on the surface of platelet membrane nanoparticles have been used to target the thrombosis site and optimize pharmacodynamics. The system reduced the potential risk of bleeding and demonstrated a broad spectrum of optimized thrombolytic therapy in three disease models: stroke, pulmonary embolism and mesenteric thrombosis. The expression of CD47 on the surfaces of erythrocytes and platelets is the main reason for the long circulatory half-life of membrane-cloaked nanoparticles [78].

## 5. Conclusions

Undesired adverse effects often occur during traditional thrombolytic therapy, including bleeding, low blood pressure and hemorrhagic stroke. To overcome these issues, a wide range of methods have been investigated to extend the circulatory half-life, improve targeting of thrombolytic agents and enhance thrombolytic efficacy. For instance, the combination of thrombolytic drugs and nanocarriers can increase the stability of the drugs in the body, avoid inactivation by antibodies and reduce clearance by organs such as the liver. Increased drug accumulation at the site of thrombosis can enhance the efficiency of therapeutics and has great potential for clinical application. Techniques to increase targeting can mainly be divided into physical methods and biological modifications. Physical responsive nano-drug delivery systems include magnetic targeting, ultrasound-mediated thrombolytic therapy and platelet-like biomimetic particles that are activated by shear stress. There are some problems with magnetic nanoparticles, such as poor dispersion and water solubility. While ultrasound therapy will disrupt the structure of the thrombus, it may also increase the risk of thrombus shedding, leading to blockage and ischemia of other vessels. Additionally, the heating effect can increase the local temperature by up to 5 °C [79], which can improve the efficiency of fibrin dissolution, but also risks damage to endothelial cells. The inertial cavitation produced by ultrasound can damage vascular endothelial cells and lead to a secondary thrombosis. High shear stress in the blood affects the interaction of vWF with GPIb on the platelet membrane surface, resulting in cross-linking of vWF with GPIb and GPIb/IIIa. Thus, high shear stress leads to platelet aggregation [80]. Platelet bionic nanoparticles are inspired by the deformation of platelets that can sense changes of blood shear stress at the site of arterial thrombi. The limitation of this shear-stress mediated targeting of platelet biomimetic nanoparticles is that they can only be used for arterial thrombi associated with very high shear stress. Compared with physical means, biologically modified nano-delivery systems have much greater biocompatibility and therefore have promise for clinical application. Antibody/ligand modification methods have much potential for improving targeting and prolonging the half-life. Membrane coating exploits cell membranes from the inherent components of a thrombus (mainly red blood cells and platelets) to coat nanoparticles. This approach allows nanoparticles to retain almost all of the natural components of the cell membrane and maintain full functionality of the cell membrane proteins. Although membrane-coated nanoparticles can significantly prolong blood circulation and improve targeting, the pattern of drug release at the target site is unknown. Antibody or peptide modification based on the components of the thrombus (activated platelets and fibrins) provides a large number of targets. All of these methods can increase the targeting of clinical thrombolytic drugs and improve efficacy, but further optimization is required. Physical and biological response targeting could be combined to provide dual-targeted precision therapy. For example, cloaking magnetic nanoparticles with cell membrane can not only reduce the toxicity of magnetic nanoparticles but also increase drug stability. Finally, we have summarized the techniques, mechanisms and limitations of various targeting methods in Table 3.

## Figures and Tables

**Table 1:** Characteristics of thrombolytic agents.

Agent	Targeting	Plasma Half-Life (min)	Immunogenicity	Ref.
UK	Non-fibrin	10–20 min	/	[34]
SK	Non-fibrin	~36 min	Immunogenic	[35]
proUK	Fibrin specific	4–6 min	/	[36]
Alteplase	Fibrin specific	4–8 min	/	[37]
Reteplase	Fibrin specific	14–18 min	/	[37]

**Table 2:** Ligand modification of liposomes.

Drug	Modification of Nanocarrier with	Target	Ref.
UK	cRGD	activated platelets	[66]
/	PPACK	thrombin	[69]
/	D7	fibrin	[70]
UK	DDmAb (D-dimer monoclonal antibody)	thrombus	[71]
SK	GSSSGRGDSPA	activated platelets	[67]
	DAEWVDVS		
tPA	CQQHHLGGAKQAGDV	activated platelets	[72]

**Table 3:** Characteristics of various targeting methods.

	Techniques	Mechanisms	Limitations
Biological response	Cell membrane	Cell membranes carry proteins such as CD47 and integrin on their surface.	The mode of drug release is unknown
	Antibody/peptide	The specific binding of antibody and antigen.	Targeting efficiency is uncertain
Physical response	Shear stress	The large shear stress at the site of arterial thrombus can destroy the structure of the nanocarrier.	It can only be applied to lesions with high shear stress
	Ultrasound	Cavitation effect between ultrasonic waves and microbubbles	Thermal effect
	Magnet	Controllable behavior of magnetic nanoparticles under a magnetic field	Safety of magnetic nanoparticles

## Data Availability

Not applicable.
